# Priority setting for the implementation of artemisinin-based combination therapy policy in Tanzania: evaluation against the accountability for reasonableness framework

**DOI:** 10.1186/1748-5908-7-18

**Published:** 2012-03-18

**Authors:** Amani Thomas Mori, Eliangiringa Amos Kaale

**Affiliations:** 1 Department of Pharmaceutics and Pharmacy Practice, School of Pharmacy, Muhimbili University of Health and Allied Sciences, Dar es Salaam, Tanzania; 2 Centre for International Health, University of Bergen, Bergen, Norway; 3 Department of Medicinal Chemistry, School of Pharmacy, Muhimbili University of Health and Allied Sciences, Dar es Salaam, Tanzania

## Abstract

**Background:**

Priority setting for artemisinin-based antimalarial drugs has become an integral part of malaria treatment policy change in malaria-endemic countries. Although these drugs are more efficacious, they are also more costly than the failing drugs. When Tanzania changed its National Malaria Treatment Policy in 2006, priority setting was an inevitable challenge. Artemether-lumefantrine was prioritised as the first-line drug for the management of uncomplicated malaria to be available at a subsidized price at public and faith-based healthcare facilities.

**Methods:**

This paper describes the priority-setting process, which involved the selection of a new first-line antimalarial drug in the implementation of artemisinin-based combination therapy policy. These descriptions were further evaluated against the four conditions of the accountability for reasonableness framework. According to this framework, fair decisions must satisfy a set of publicity, relevance, appeals, and revision and enforcement conditions.

In-depth interviews were held with key informants using pretested interview guides, supplemented with a review of the treatment guideline. Purposeful sampling was used in order to explore the perceptions of people with different backgrounds and perspectives. The analysis followed an editing organising style.

**Results:**

*Publicity*: The selection decision of artemether-lumefantrine but not the rationale behind it was publicised through radio, television, and newspaper channels in the national language, Swahili. *Relevance*: The decision was grounded on evidences of clinical efficacy, safety, affordability, and formulation profile. Stakeholders were not adequately involved. There was neither an appeals mechanism to challenge the decision nor enforcement mechanisms to guarantee fairness of the decision outcomes.

**Conclusions:**

The priority-setting decision to use artemether-lumefantrine as the first-line antimalarial drug failed to satisfy the four conditions of the accountability for reasonableness framework. In our understanding, this is the first study to evaluate priority-setting decisions for new drugs in Tanzania against the accountability for reasonableness framework. In addition to the demand for enhanced stakeholder involvement, publicity, and transparency, the study also calls for the institution of formal appeals, revision, and regulatory mechanisms in the future change of malaria treatment policies.

## Background

Malaria case management with pharmaceuticals is a major challenge in many malaria-endemic countries. Old but cheap and effective medicines are increasingly facing resistance. By 2009, nearly all *Plasmodium falciparum *malaria-endemic countries, most being in sub-Saharan Africa, had changed their treatment policies to artemisinin-based combination therapies [1,2]. On the other hand, rapidly increasing pharmaceutical expenditure poses another major obstacle and has led to an increased need for prioritisation in pharmaceuticals worldwide [3,4]. When Tanzania changed its National Malaria Treatment Policy in 2006, three choices of artemisinin-based combination therapies recommended by the World Health Organisation (WHO) were available [5]. The guiding principle was to guarantee access to antimalarial drugs that are safe, affordable, effective, acceptable, and of good quality for patients infected with malaria [6]. Limited resources, especially for publicly funded healthcare systems, mean that priorities must be set, since not all the available interventions can be provided for all who need them [7]. Among the available alternatives, artemether-lumefantrine (ALu) was prioritised to be the first-line drug for the management of uncomplicated malaria to be available at a subsidized price in both public and faith-based healthcare facilities.

Tanzania has a fairly well-distributed healthcare system, within which 66% of all the healthcare facilities are owned by the government [8]. About 80% of the population has access to healthcare services that, to a large extent, do not meet the minimum acceptable standards [9]. In 1993, the government introduced a cost-sharing policy and health insurance schemes to complement government financing and as an attempt to improve the quality of services [10]. Currently, about 15% of the population is covered by health insurance [11]. Households contribute 47% of healthcare financing through out-of-pocket expenditure and 43% is financed by the government and donor support [12].

Rational malaria treatment policy formulation presents challenges to many endemic countries. At times, donors may favour an approach that conflicts with the national preferences [13,14]. Studies on the previous policy change in Tanzania, from chloroquine to sulphadoxine-pyrimethamine, described the process as irrational. It was characterised by disagreements among the stakeholders, mistrust of evidence, and accusations of influence from pharmaceutical companies [15,16]. The change of policy was influenced by only a few individuals who were involved in various ways with malaria [16].

### Accountability for reasonableness and its applications in Tanzania

The accountability for reasonableness framework (AFR) (Table 1) is a procedural framework that was developed as an approach to guarantee fairness and legitimacy of priority-setting decisions in health [17-19]. When properly implemented, AFR can prevent the development of fairness and legitimacy problems grounded on mistrust of the processes. The framework outlines a roadmap of four key conditions that must be fully fulfilled in the decision-making process. In recent years, despite its limited application in developing countries, AFR has been gaining popularity in Tanzania, especially at the district level [20-22].

**Table 1:** Accountability for reasonableness framework

Condition	Description
Publicity	Decisions regarding both direct and indirect limits to care and their rationales must be publicly accessible. The process of priority setting must be open and transparent and consultations and public hearings should be held. Publicity and involvement of key stakeholders are particularly important in contexts where policy and programmatic decisions occur in a multi-actor environment and affect large parts of the population.
Relevance	The rationales for priority-setting decisions should aim to provide a reasonable explanation of how organisations and health providers seek to meet the health needs of a population under reasonable resource constraints. Specifically, a rationale will be reasonable if it appeals to evidence, reasons, and principles that are accepted as relevant by fair-minded people who are disposed to finding mutually justifiable terms of cooperation. By 'fair-minded' people, we do not simply mean our friends or people who just happen to agree with us.
Appeals and revision	There must be mechanisms for challenge and dispute resolutions regarding priority-setting decisions, and, more broadly, opportunities for revision and improvement of policies in the light of new evidence or arguments.
Enforcement	There must either be a voluntary or public regulation of the process to ensure the first three conditions are met.

District and regional health planners have applauded the framework for its ability to enhance participation, transparency, and the development of relevant criteria for decision making [20]. Where it has been used to evaluate priority-setting decisions, it facilitated the identification of key areas that needed improvement [21,22]. Daniels and Sabin, who developed the framework, suggest that 'accountability for reasonableness' is relevant to pharmaceutical priority setting:

Managing access to pharmaceuticals is a microcosm of the limit-setting problems of healthcare systems as a whole. If we are right that accountability for reasonableness is a solution to the legitimacy problem in health systems as a whole, then it should be possible to illustrate what such accountability would mean in practical terms... --[17].

This is the first study to apply the AFR framework to evaluate priority-setting decisions that involve selection of drugs in Tanzania. The selection process for drugs to be listed on the national essential medicine list also involves priority setting. We argue that evaluation of these decisions against the AFR framework is one method of improving the process of drug selection. This study therefore reports on the evaluation of the priority-setting decision for the implementation of artemisinin-based combination therapy policy against the four conditions of the AFR framework.

## Methods

### Study type and participants

This was a qualitative study in which in-depth interviews were held with key informants. At least two participants were selected from the following list of institutions: Muhimbili National Hospital (MNH), National Malaria Control Programme (NMCP), WHO office in Tanzania, Ministry of Health (MoH), Tanzania Food & Drug Authority (TFDA), Medical Stores Department (MSD), and Muhimbili University of Health and Allied Sciences (MUHAS). Participants were experts on malaria research, administration of public health institutions, management of patients, and regulation and management of pharmaceuticals.

### Sampling and sample size estimation

The purposive sampling method was used to select the key informants from the acknowledgements list of the treatment guideline. A sample of 15 participants was selected based on Guest et al.'s recommendations, which imply that, in a piece of research that is narrow in scope and has a homogenous sample, data saturation is achieved after 12 interviews when thematic analysis is employed [23].

### Data collection

Data were collected by in-depth interviews held with key informants using pretested interview guides and reviews of the treatment guideline between May and July 2010. A digital audio recorder was used to record the conversations. The in-depth interview guide consisted of questions corresponding to the AFR framework [17]. Each interview lasted for about one hour. The four conditions of the framework were the main themes. The accessibility of the decision and its rationales were categorised under publicity. Rationales and stakeholder involvement were categorised under relevance. Efficacy, affordability, availability, and cost effectiveness were subcategorised under rationales. Representation, existence of explicit procedures for stakeholder involvement in the task force committee, and consultations were subcategorised under stakeholder involvement.

### Data management and analysis

Interviews were transcribed and then analysed using the ATLAS.ti (ATLAS.ti Scientific Software Development GmBH, Berlin, Germany), a qualitative data analysis (QDA) software, using an editing organising-style method [24]. First the template was prepared based on the interview guide, according to Kvåle's analysis approach [25]. This approach involves the defining of codes before an in-depth analysis of the data. Meaningful units or segments of text in the transcripts and the treatment guideline that correspond to the template or related to this study were identified. Networks for each code with their corresponding quotations were formulated using the QDA program. These networks enabled the researchers to analyse the descriptions of each participant for each code for the transcribed data. Thereafter, memos were written to combine these descriptions with those that emerged from similar codes in the treatment guideline. A network of all codes and their corresponding memos under their subcategories was made, and this enabled the researchers to analyse the descriptions in the memos and reach a conclusion for each subcategory. Finally, a network of the main themes of fairness with their descriptions was made for final analysis and evaluation, in order to determine whether or not the four conditions were satisfied.

### Data validity

To ensure the validity of the data, participants were assured of the aim of the study and their names were anonymised. The in-depth interviews were held in places where interviewees felt comfortable, and the conversations were recorded by using a digital audio recorder. Interviews were transcribed by the interviewer shortly after each session. Telephone numbers of the interviewees were taken so that if any information was missing, they could be called for clarification. Transcripts were read and re-read several times so that the researcher could become familiarised with their contents before analysis. Coding of both the transcripts and the treatment guideline were done separately, and the emerging descriptions were triangulated during memo writing.

### Ethical issues

Ethical clearance (Ref. No. MU/PGS/SAEC/Vol. III/185) was provided by the MUHAS Ethical Review Committee. Participants were asked for verbal informed consent and for permission to be recorded prior to the interviews.

## Results

### Publicity condition

#### Accessibility of the decision and its rationales

A majority of the participants reported that the decision about the selection and use of ALu was disseminated to the public by the use of radio, television, and newspaper channels in the national language, Swahili. Meetings with other stakeholders, such as pharmacists, were also held as a means of disseminating this information. They also reported that all the public healthcare facilities from the dispensary to the regional hospital level received the treatment guidelines, which contained the decision and rationales for the prioritisation of ALu.

### Publicity condition: evaluation

The decision about the selection of ALu, but not the reasons behind it, was communicated through advocacy campaigns by National Malaria Control Program (NMCP) and Ministry of Health (MoH) using different media channels in Swahili, the national language. The rationales were written in the treatment guidelines, which are not easily accessible to the public, patients, or other stakeholders who were not directly involved with the task force [26].

### Relevance condition

#### Rationales

##### Efficacy

Participants reported that the problem of parasite resistance to sulphadoxine-pyrimethamine was the major factor that caused the change of the National Malaria Treatment Policy. At the time of this policy change, three choices of artemisinin-based combination drugs recommended by WHO for management of uncomplicated malaria were available. These drugs included sulphadoxine/pyrimethamine-artesunate (SP-AS), amodiaqine-artesunate (AQ-AS), and ALu. ALu had the following advantages over the other drugs: (1) it was the only fixed combination drug among the three, (2) neither of its components had documented reports of parasite resistance, (3) it was safer, as amodiaquine's safety profile had already generated concern among healthcare providers and the general public and (4) it was the most efficacious drug.

###### Affordability

Participants reported that affordability was not discussed because of the agreement between the government and the Global Fund to provide the drug at a subsidized price at the public and faith-based healthcare facilities. This was facilitated by the agreement between WHO and the company that manufactures ALu, which made the drug available at cost price for use in the public sector for malaria-endemic developing countries. However, some participants raised concerns about the sustainability of this financing approach.

... as a country you cannot just sit and wait for somebody to carry the burden of maintaining your population's health, imagine if Global Fund does not keep on sustaining this program!--Representative from MSD

##### Availability of subsidized artemether-lumefantrine

Participants pointed out that, based on Global Fund requirements, the subsidized ALu could only be made available at public and faith-based primary to tertiary healthcare facilities. One participant who works at a tertiary hospital expressed concerns about the low availability of ALu in the primary healthcare facilities:

I receive patients from the dispensaries who have been given sulphadoxine-pyrimethamine and amodiaquine and they sometimes ask why they are still prescribing these drugs when the first-line drug is artemether-lumefantrine!--Physician from MNH

One participant was concerned about the urban-poor populations who have to make a choice between long waiting times and unreliable public health services versus reliable but unaffordable services in the private sector. Participants from National Malaria Control Program (NMCP) said that other strategies were being considered to make sure ALu was also available at a subsidized price in the private-for-profit healthcare facilities.

##### Effectiveness and cost-effectiveness

Participants said that fixed-combination antimalarial drugs have better compliance, hence are more effective compared to non-fixed combinations. However, artemether-lumefantrine's six-dose regimen of four tablets to be taken twice a day with a fatty meal for three successive days was a major concern.

There are obvious improvements, admissions due to severe malaria nowadays are few and this could be explained by the effectiveness of artemether-lumefantrine. As a tertiary hospital we used to have a lot of patients coming in coma due to cerebral malaria; this is not research data but my own observation.--Physician from MNH

About its cost-effectiveness, the majority of participants reported that there were no research data at the time of the policy change that indicated the cost -effectiveness of ALu.

#### Stakeholder involvement

##### Representation and existence of explicit procedures for stakeholder involvement

The task force committee was composed of 25 members; 21 were medical doctors and four were pharmacists. The medical doctors were paediatricians, obstetricians/gynaecologists, parasitologists, and pharmacologists from Muhimbili National Hospital (MNH) and Muhimbili University of Health and Allied Sciences (MUHAS). Others were working with the WHO country office, National Malaria Control Program (NMCP) and Integrated Management of Childhood Illnesses (IMCI) programs within Ministry of Health (MoH). The pharmacists were working with Medical Store Department (MSD) in the procurement and distribution of drugs, Tanzania Food and Drug Authority (TFDA) in food and drug regulations, and the pharmaceutical services unit at the Ministry of Health. All the committee members were from institutions based in the city of Dar es Salaam. Participants from NMCP who coordinated the policy-change process reported that they did not have written documents on procedures to select the committee members but that they 'knew' the main stakeholders. This approach was criticised by one of the participants:

Sometimes I feel as if it is not adequate because if I had not contacted those people with my findings, I might not have been invited. So there might be other people who are in the field but because they had not made contact with Ministry of Health officials, they did not get the opportunity to be part of the task force committee.--Researcher from MUHAS

Participants reported that patients and the public were indirectly involved through representation and consultations. It was also argued that the task force committee members were potential malaria patients, and therefore, they were themselves representatives of patients and the public. The idea of direct patient and general public involvement in the committee was opposed by some participants who labelled it as inappropriate because policy making is an expert's field.

##### Consultations

Participants reported that many consultations were held before and even after the assembly of the task force committee to get input and technical advice about policy change. Participants from National Malaria Control Program (NMCP) said that the first draft of the guideline was reviewed by experts from the National Institute for Medical Research, Malaria Consortium-Kampala, WHO Afro, Médecins sans Frontières, Ifakara Health Institute, and Eduardo Mondlane-Maputo (Mozambique). They also reported that the draft guidelines were shared by all district and regional medical officers as well as partner countries for technical advice. Councils gave input during the zonal training sessions that were conducted countrywide.

### Relevance condition: evaluation

#### Rationales

The reasons for the prioritisation of ALu appeal to stakeholders because they were based on scientific evidence of clinical efficacy, safety, and formulation profiles. Financing mechanisms were already in place to ensure that the drug would be available and affordable at the public and faith-based healthcare facilities.

#### Stakeholder involvement

The study found that there were no transparent procedures to engage the stakeholders. Some stakeholders were involved because of their positions in the relevant institutions, while others were involved through informal links with officials in the Ministry of Health (MoH) or National Malaria Control Program (NMCP). The committee lacked professional, institutional, and countrywide representation. It was largely made up of medical doctors and a few pharmacists; neither nurses nor medical lab personnel were included. All the physicians, specialists, and academicians within the task force committee came from one hospital, i.e. Muhimbili National Hospital (MNH) and one university, Muhimbili University of Health and Allied Sciences (MUHAS). There were other similar institutions in the country that could have provided members to increase representation.

### Appeals and revision condition

Participants said that it is not easy for anybody to appeal against the policy; however, if established facts and evidence on toxicities and level of resistance were to be provided, the policy decision could be revised.

I think the best way for people to appeal is to provide scientific data in the scientific forum; it is impossible for an individual to come up and say this is not the right drug. From the community side, I think the only mechanism they have is to express their concerns in the newspapers and other media; otherwise, I don't see any other channels at the moment.--Participant from TFDA

Pharmacovigilance forms, which are used as post-marketing surveillance tools in monitoring drug safety, were mentioned as effective tools to generate evidence on the safety profile of ALu. However, one participant disagreed with the reliability of this approach.

Most of our clinics are crowded, before you can take a minute or two to fill in the pharmacovigilance form, 20 people are already shouting at you, so it is one avenue but is not satisfactory.--Physician from MNH

### Appeals and revision condition: evaluation

There is no mechanism in place to appeal against the decision to prioritiseALu.

### Enforcement condition

There was no enforcement mechanism. The demand for enforcement was seen as new and was considered unnecessary by some participants. Other participants said that an independent enforcement body is necessary during a treatment policy change.

I am not sure if there was a watchdog. I think it is really very important because sometimes people say the drug companies are pushing for policy change.--Pharmacologist from MUHAS

### Enforcement condition: evaluation

Despite growing concerns about the influence of pharmaceutical companies on change of treatment policies, we did not find any enforcement mechanism to ensure the other three conditions were met.

## Discussion

This study evaluated the process of changing the Tanzania National Malaria Treatment Policy against the AFR framework. The process resembled other priority-setting decisions, in that it involved making choices among competing alternatives in the context of limited resources, conflicting evidence, diverse stakeholder involvement, and the vagaries of political cycles [27]. The influence of power was evident insofar as some individuals, groups, and institutions were better positioned than others to influence decision outcomes. Power differences were associated with professional and institutional status, the reliability of research findings, ownership of financial resources, and the historical dominance of the public healthcare delivery system.

When evaluated against the AFR, this study found that the publicity condition was not fully satisfied. The study found that the decision to prioritiseALu was made public, but not the reasons behind it. Publicity requires that the decision and its rationales, such as the ones for coverage of new drugs like ALu, be publicly accessible to all stakeholders, including the patients [17]. Similar findings have been reported at the district level, where again the decision but not the reasons behind it were communicated to stakeholders [21].

The study found that the rationale but not the stakeholder involvement part of the relevance condition was satisfied. ALu was the only artemisinin-based antimalarial drug that was in a fixed-combination formulation. It was also the most efficacious drug and neither of its two components had any documented reports of parasite resistance [6]. Cost-effectiveness evidence was not used to inform the decision, but studies have shown that ALu was the most cost-effective drug among the available alternatives [28,29]. The selection of ALu was influenced by an agreement between the WHO and Novartis, the company that manufactures ALu, to monitor the global demand and supply of the drug. In exchange, this agreement enabled malaria-endemic developing countries to procure the drug at cost price for distribution in the public healthcare facilities [2]. However, the major concern among the stakeholders is the sustainability of the existing financing arrangement. Affordable Medicines Facility-Malaria (AMFm) is currently pioneering a pilot study to involve the private for-profit facilities [30] because it has been shown that the private sector plays a major role in malaria case management [31,32]. These attempts to increase access to artemisinin-based antimalarials must ensure that the delivery, financial, and governance arrangements are compatible with the national context needs [33]. Although artemisinin-based antimalarial drugs are the best hope for malaria case management, there are various challenges facing their use. In areas where ALu has been deployed, patients frequently do not adhere to its six-dose regimen, hence compromising its effectiveness [34]. Studies have also shown that many doses of these expensive drugs are lost due to symptomatic diagnosis and inadequate slide reading [35-37].

The study found that stakeholders were not adequately involved and represented. Appropriately conducted stakeholder participation not only facilitates and widens discussions in the decision-making process but also enhances publicity [17]. The task force committee was also not representative. Proper representation of healthcare professionals who also represent different institutions at the discussion table was necessary. This would have complemented the findings of the IMPACT Project, which was carried out in order to inform the policy change decision [38], which ultimately would have enhanced accountability for reasonableness.

Even though we argue in favour of wide professional and institutional representation, this should not aim to replace patients and the general public. There is evidence that health professionals' views can differ considerably from those of their patients [39]. However, the involvement of the public and patients in an area populated by professionals is a challenge, especially in developing countries. It has been shown that potential stakeholders at the district level in Tanzania have no knowledge, skills, and experience to be able to participate effectively in priority-setting decisions [20]. Use of large-scale surveys and the rapid appraisal method have been tested and have proved successful in developing countries. The views of key groups of people, like teachers, shop owners, and students, elicited through interviews can adequately represent the opinions of their communities [40].

The appeals and revision condition was not satisfied. To our knowledge, there are no formal appeals mechanisms in the Tanzanian healthcare system for policy decisions relating to access to new drugs or healthcare. Pharmacovigilance systems in developing countries are weak, and hence cannot be considered an efficient appeal mechanism to inform policy makers of a drug causing harm to its users. The lack of reliable appeal mechanisms suggest that good arguments and evidences against the original decision and which aim at improving faulty policy decisions can have no clear way back onto the decision table. A study by Maluka et al. reported a lack of clear formal channels for appeals at the district level in Tanzania [21].

The enforcement condition was not satisfied. This condition recognises that public or private regulation may be necessary if self-regulation proves unsuccessful or inadequate. There are growing concerns about the influence of pharmaceutical companies, which lobby and push for national treatment policy changes, especially in developing countries [14,16]. Hence, the establishment of an independent regulatory body to guarantee fair process during policy change is necessary. Other studies in Tanzania have also reported lack of enforcement mechanisms for priority-setting decisions due to poor organisation, unclear lines of authority, low level of public awareness, and inadequate public enforcement mechanisms [21,22].

### Strengths and limitations of the study

The qualitative research method is the best approach to study complex phenomena when a researcher wants to capture the views of individual persons, including their feelings, understandings, interpretations, beliefs, and ideologies. Through the use of this method, the participants shared their stories and conflicting views beyond what the researchers expected to hear or what is available in the literature.

The study used the AFR as an evaluation framework; however, there are objections to its use as an approach to achieve justice and fairness [41,42]. Moreover, differences may exist between the AFR's definition and participants' views on the concept of fairness and its evaluation. The results obtained from the interviewed participants may not be the general views of the whole group, as we interviewed only a part of the task force team who may not be the true representation of the group. As emerged from the interviews, some decisions were made before the assembly of the task force committee, while other decisions were not made by the task force committee per se. Due to resource and time constraints, we did not interview any individuals from the consultation group, the general population, or patients' representatives. Other stakeholders include healthcare professionals outside the task force committee, the ALu manufacturing company, and Global Fund as the financing institution. We strongly believe that this study is missing the views of other individuals who may possess important and reliable information related to this study.

## Conclusions

This study applied the AFR framework to evaluate the priority-setting decision for new antimalarial drugs at the national level. The study found that the priority-setting decision in favour of ALu failed to satisfy the four conditions of fair process prescribed in the ethical framework of accountability for reasonableness. The findings of this study are consistent with the reports of other studies that evaluated priority-setting decisions against AFR at the district level. The study emphasises the need to involve a wide range of stakeholders in priority-setting decisions. It further highlights the need to make priority-setting decisions and their rationales public and to establish mechanisms for appeal, revision, and regulation in order to enhance fairness and legitimacy of the decision outcomes.

## Competing interests

The authors declare that they have no competing interests.

## Authors' contributions

ATM conceived and designed the study, collected the data, performed the analysis, and prepared the draft of the manuscript. EAK fine-tuned the study design and coordinated the data collection and analysis. All authors read and approved the final manuscript.
